# Network Analysis of Inflammatory Genes and Their Transcriptional Regulators in Coronary Artery Disease

**DOI:** 10.1371/journal.pone.0094328

**Published:** 2014-04-15

**Authors:** Jiny Nair, Madankumar Ghatge, Vijay V. Kakkar, Jayashree Shanker

**Affiliations:** 1 Mary and Garry Weston Functional Genomics Unit, Thrombosis Research Institute, Bengaluru, Karnataka, India; 2 Tata Proteomics and Coagulation Unit, Thrombosis Research Unit, Bengaluru, Karnataka, India; 3 Thrombosis Research Institute, Bengaluru, Karnataka, India; 4 Thrombosis Research Institute, London, United Kingdom; Nazarbayev University, Kazakhstan

## Abstract

Network analysis is a novel method to understand the complex pathogenesis of inflammation-driven atherosclerosis. Using this approach, we attempted to identify key inflammatory genes and their core transcriptional regulators in coronary artery disease (CAD). Initially, we obtained 124 candidate genes associated with inflammation and CAD using Polysearch and CADgene database for which protein-protein interaction network was generated using STRING 9.0 (Search Tool for the Retrieval of Interacting Genes) and visualized using Cytoscape v 2.8.3. Based on betweenness centrality (BC) and node degree as key topological parameters, we identified interleukin-6 (IL-6), vascular endothelial growth factor A (VEGFA), interleukin-1 beta (IL-1B), tumor necrosis factor (TNF) and prostaglandin-endoperoxide synthase 2 (PTGS2) as hub nodes. The backbone network constructed with these five hub genes showed 111 nodes connected via 348 edges, with IL-6 having the largest degree and highest BC. Nuclear factor kappa B1 (NFKB1), signal transducer and activator of transcription 3 (STAT3) and JUN were identified as the three core transcription factors from the regulatory network derived using MatInspector. For the purpose of validation of the hub genes, 97 test networks were constructed, which revealed the accuracy of the backbone network to be 0.7763 while the frequency of the hub nodes remained largely unaltered. Pathway enrichment analysis with ClueGO, KEGG and REACTOME showed significant enrichment of six validated CAD pathways - smooth muscle cell proliferation, acute-phase response, calcidiol 1-monooxygenase activity, toll-like receptor signaling, NOD-like receptor signaling and adipocytokine signaling pathways. Experimental verification of the above findings in 64 cases and 64 controls showed increased expression of the five candidate genes and the three transcription factors in the cases relative to the controls (p<0.05). Thus, analysis of complex networks aid in the prioritization of genes and their transcriptional regulators in complex diseases.

## Introduction

Coronary Artery Disease (CAD) is a chronic inflammatory disease. There is ample evidence on the role of inflammation in all stages of the atherosclerotic disease process [1], [2]. Genetic studies have revealed many causal or susceptible inflammatory loci associated with CAD, the manifest form of atherosclerosis [3], [4]. Circulating inflammatory biomarkers such as C-reactive proteins (CRP) and certain cytokines are also elevated in acute coronary syndrome, which reflect the extent of myocardial necrosis and ischemia/reperfusion damage [5]. All these studies singularly demonstrate that inflammatory genes act in an interactive manner to orchestrate the disease associated risk. In most conditions, inflammation signaling show a cascade effect with some molecules acting as primary triggers that stimulate a secondary line of molecules and so on, eventually generating a strong inflammatory milieu. Identification of such key inflammatory targets is critical from a translational aspect in order to treat the ‘inflammation’ component of the disease that can lead to slowing down or even arrest in its pathological and clinical progression.

In this regard, systems biology focuses on understanding the complex nature of CAD by integrating the information across various systems such as the genome, transcriptome, proteome and the metabolome, which is in direct contrast to traditional approaches that focus on individual genes, proteins or metabolites [6], [7]. Despite decades of genetic research on CAD, it is striking to note that the identified genetic loci explain only a small proportion of the disease heritability [8], suggesting that there may still be many other genes involved in CAD that remain unknown to date. Network based approach is being extensively used for the prediction of putative candidate genes, prioritizing drug targets [9] and in the construction of gene regulatory networks [10] thus utilizing the data from gene expression or genome wide linkage and association studies. This approach primarily focuses on the inter-relationship between the various components using protein-protein interaction (PPI) network and assist in the identification of the best discerning molecules associated with the disease. Studies have employed a PPI network-based approach to identify important novel cardiovascular disease genes [11], [12]. There is growing interest in combining genome wide association studies with network analysis to improve our understanding of the molecular basis of complex diseases [13], [14]. Feldmann et al. and more recently Björkegren et al utilized this combined approach to delineate the transcription factor- regulatory molecules that potentiate the key target genes in atherosclerosis [15], [16]. Motivated by these studies and to overcome the inherent limitations in the selection of genes for network construction, we have taken into consideration all the inflammatory genes implicated in various inflammatory diseases and systematically analyzed the putative causal genes and deregulated pathways. Thus, the present study enumerates the following work: i) identify key inflammatory genes in CAD derived from an inflammatory bionetwork, generated with the help of bioinformatics tools; ii) perform functional enrichment analysis to ascribe biological functions to the key genes; iii) identify key transcriptional regulators; and iv) study the whole blood gene expression levels of these regulators in a matched cohort of CAD patients (cases) and controls. The study findings provide interesting evidence of increased expression of key candidate genes and their transcriptional regulators among the cases, thus enabling us to prioritize the significant contributors of inflammation among a myriad of inflammatory biomarkers through the application of computational biology. Given the simplicity of the methodology involved, this framework can be easily extended to other complex diseases.

## Materials and Methods

**Figure 1:**
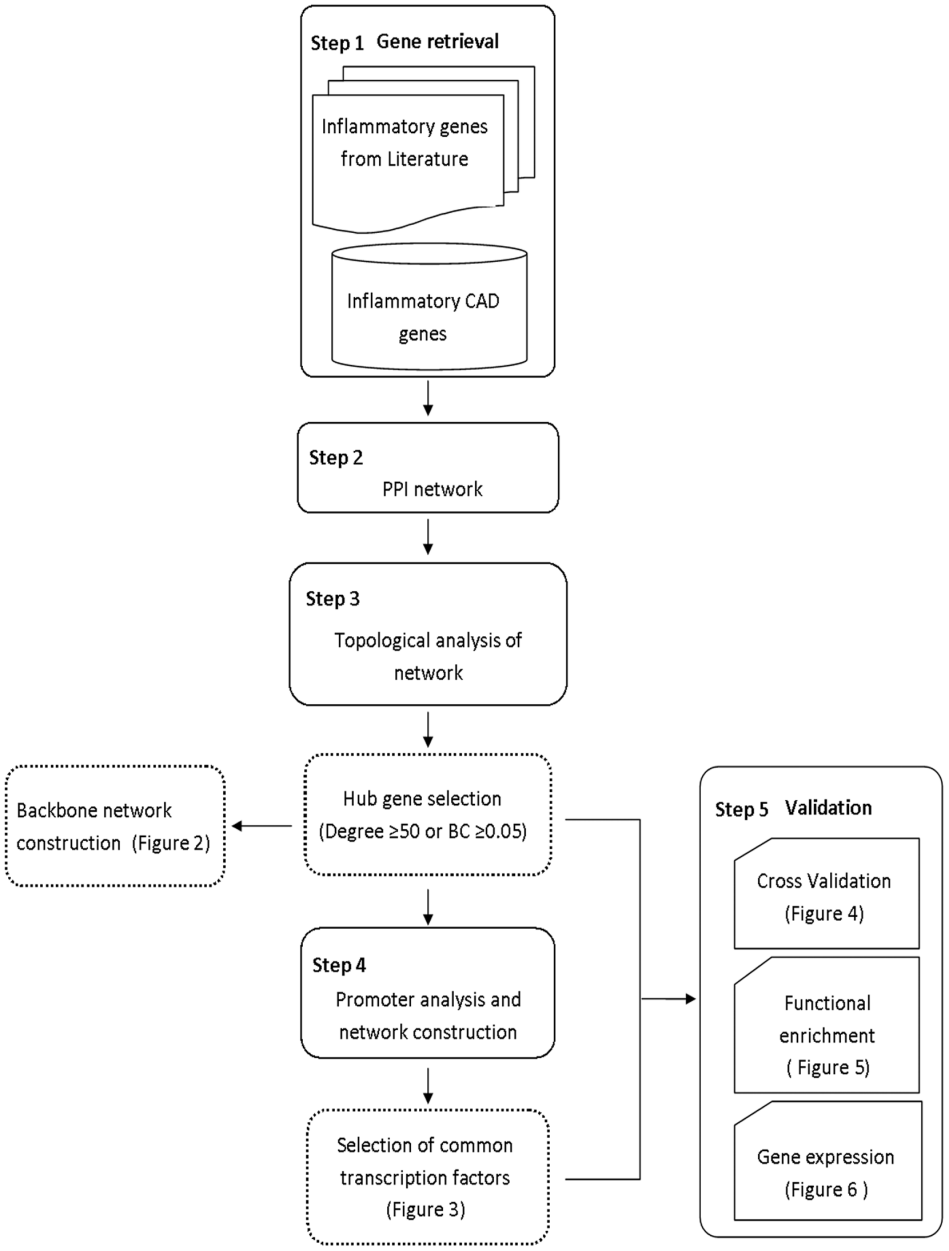
An overview of the work flow. An overview of the work flow has been summarized and consists of the following steps- Step 1: Retrieval of inflammatory genes from literature using *PolySearch* text mining tool and from *CADgene* database; Step 2: Analysis of protein interactions using *STRING* database; Step 3: Topological analysis of network using *Cytoscape v2.8*, based on betweenness centrality and node degree, leading to the identification of hub genes and construction of the backbone network; Step 4: Construction of a regulatory network for the hub genes and identification of common transcription factors (TFs) regulating them; Step 5: Validation of the hub genes based on cross-validation (1 to 4 node deletion), functional enrichment analysis (ClueGo) and quantitative evaluation of key genes and their common transcription factors by real-time PCR.

The general schematic approach of the study is summarized in Figure 1.

### Gene retrieval and prioritization

Genes associated with inflammation in general and CAD in particular was used for the construction of the inflammatory bionetwork. These genes were retrieved from two independent sources, namely *PolySearch* and *CADgene* database. PolySearch is a comprehensive text mining system that extracts the relationships between diseases, genes, mutations, drugs and metabolites in humans from different types of biomedical text databases such as *PubMed, OMIM, DrugBank, SwissProt, Human Metabolome Database* (HMDB), *Human Protein Reference Database* (HPRD) and *Genetic Association Database* (GAD) [17]. The search was performed using ‘Disease-Gene/Protein Association’ for query type and ‘Inflammation’ as the query keyword, which resulted in a list of 589 genes. This initial gene list was prioritized based on a relevancy score, expressed as Z score, and refers to the number of standard deviations of the relevancy score above the mean value. A higher Z score denotes a lesser likelihood that the outcome is due to chance. We selected genes having a Z score >1 and arrived at a final list of 78 genes.

Next, we selected the inflammatory genes from CADgene database [18], which includes ∼300 genes curated from literature and categorized according to their functionality. This search yielded 68 genes. The lists of genes obtained from the two databases were then combined and the redundant gene symbols were removed, to finally obtain 124 genes (Table S1). We then searched for disease terms associated with the 124 putative genes using *FunDO* [19], which suggested that 70% (87 genes out of 124) of the genes were associated with CAD and belonged to the following disease ontology (DO) terms: atherosclerosis, multiple sclerosis, intermediate coronary syndrome, heart failure, ischemia, cardiovascular disease, heart disease and vascular disease.

### Protein-Protein interaction (PPI) network

The 124 genes listed in Table S1 were considered as the seed molecules from which we obtained direct and indirect protein-protein interactions using *STRING 9.0* database (Search Tool for the Retrieval of Interacting Genes) [20]. This database provides information on both experimental and predicted interactions from varied sources based on their neighborhood, gene fusions, co-occurrence, co-expression, experiments and literature mining. We constructed an extended network based on a high confidence score of 0.7, which implies that only interactions with high level of confidence were extracted from the database and considered as valid links for the PPI network.

### Construction and analysis of the network

The protein-protein interaction (PPI) network was visualized using *Cytoscape v 2.8.3* software. The network was analyzed based on topological parameters like betweenness centrality (BC) and node degree using a Cytoscape plug-in called ‘*Network Analyzer’* [21]. In a given network, each gene is represented as a node and the interactions between the nodes are defined as edges. Degree indicates the number of edges linked to a given node and nodes having high degree may represent the hub genes possessing important biological functions. Betweenness centrality reflects the importance of the node based on the number of shortest paths that pass through each node. The final network was visualized based on these parameters wherein we mapped the node degree to the node size and betweenness to the node color in the network view. Nodes having high degree were displayed as a big circle while shades of red to green color represented high to low BC values for the node [21]. In short, we considered nodes carrying high degree and betweenness centrality to be the hub genes.

### Construction of the regulatory network

We carried out promoter analysis using *MatInspector* program from *Genomatix* software suite (Genomatix, Munich, Germany) [22] to identify transcription factors (TFs) that regulate the hub genes. Transcription factors which showed evidence of more than one binding site was selected for further analysis. Functionally related or similar TFs were grouped into the same family. Following the selection of regulators for the seed genes, we constructed the regulatory network using *Cytoscape*. Transcription factors that showed high connectivity with the target genes were then selected for assessing their expression levels in the matched cases and controls.

### Validation of hub genes

In order to evaluate the accuracy of our backbone network and determine the frequency of hub genes, we constructed test networks using a part of the 124 seed genes. The test networks were constructed by leaving out 1 to 4 genes by repeated removal of the top 22 nodes that had the highest BC and node degree. Deletion of one node each time would result in the construction of 22 networks. However, if the number of omitted genes was more than 2, we would have had a large number of combinations. Therefore, deletion of more than one node was carried out five times by omitting one hub gene each time and randomly selecting the remaining genes from the top 22 nodes. This step was carried out for all the hub genes, namely IL-6, TNF, IL1B, VEGFA and PTGS2. Deletion of 2, 3 or 4 nodes resulted in the construction of 25 networks. Finally 97 (22+3*25) test networks were constructed and analyzed using *Cytoscape v 2.8.3*. The detailed list of nodes omitted for each test network is provided in Table S3. Eventually, the top 5 nodes were determined in these 97 test networks. The accuracy of the network, which is the proportion of the hubs retained in the test network to the number of hubs (n = 5) in the main network, was finally measured.

### Functional enrichment analysis

To further understand the biological relevance of the hub genes and their regulators in CAD, we performed functional enrichment analysis using *ClueGO* [23]. *ClueGO* facilitates the visualization of functionally related genes displayed as a clustered network and chart. The statistical test used for the enrichment was based on right-sided hypergeometric option with a Benjamini-Hochberg correction and kappa score of 0.3.

### Testing the mRNA expression of genes constituting the backbone network and core regulatory molecules by real-time qPCR

Potential candidate genes and their common regulators obtained from the network analyses were selected for mRNA expression assay by quantitative RT-PCR (real time-PCR or qPCR) in 64 CAD individuals and 64 matched controls.

#### Study population

Two groups consisting of CAD patients (n = 64) and age and gender-matched controls (n = 64) were selected from the Indian Atherosclerosis Research Study (IARS). Detailed design of the IARS has been published [24]. Briefly, the IARS is an ongoing epidemiological study with an objective to investigate the genetic factors and biomarkers against a backdrop of the conventional cardiovascular risk factors in Asian Indians living in India. Recruitment of cases and controls was based on predefined inclusion/exclusion criteria. CAD patients were included if they belonged to any of the following criteria: i) angiographically confirmed presence of CAD with >70% stenosis in any one major epicardial artery or >50% stenosis in two or more arteries, ii) having past history of myocardial infarction, and iii) undergone/undergoing percutaneous transluminal coronary angioplasty or bypass graft surgery.

Healthy volunteers who were clinically asymptomatic for CAD and other inflammatory disorders, enrolled from the same geographical area as that of the proband, having no family history of cardiovascular disease and showing normal ECG readings were treated as controls. Informed consent was obtained from all the study participants. The study has been approved by the Institutional Ethics Committee and follows the bioethical guidelines of the Indian Council of Medical Research (ICMR) [25].

#### Clinical and Biochemical assessment

Detailed demographics and anthropometric measures were recorded for each participant based on a personal interview. Presence of hypertension and diabetes was ascertained based on self-report of physician's diagnosis and/or use of prescription medications along with perusal of their medical records. Body mass index (BMI) was calculated as body weight (kg) divided by the square of height (m^2^). Prevalence of metabolic syndrome (MS) in the cohort was assessed based on the modified Adult Treatment Panel III(ATP-III) criteria which includes lower cut-offs for waist circumference(WC) (≥90 cm for men and ≥80 cm for women) and appears to be a better criteria for classification of MS in Asian Indians [26].

Venous blood collected after overnight fasting of 12 to 14 hrs was centrifuged to separate the serum, EDTA and plasma samples. Plasma levels of total cholesterol (TC), triglyceride (TG) and High-density lipoprotein-cholesterol (HDL-c) were measured using Siemens Dimension Flex reagent cartridge (Siemens Healthcare Diagnostics Ltd, UK) and standards from Randox laboratories (Crumlin, UK) and assayed on Siemens dimension Xpand plus instrument (Siemens Dade Behring, Liederbach, Germany).

#### Real-Time QPCR Assay setup and analysis

Total RNA was isolated from whole blood cells using QIAamp RNA Blood mini kit (Qiagen Inc, Hilden, Germany) and reverse transcribed to cDNA using cDNA archive kit (Applied Biosystems Inc., Foster Coty, CA, USA), following manufacturer's instructions. Quantitative RT-PCR was performed in duplicate using SYBR green chemistry on 7900 HT Fast RT-PCR system (Applied Biosystems, Foster City, CA). Relevant primer pairs were selected from the PrimerBank [27] and verified using BLAST search. The primer sequences are listed in Table S2. The efficiency of RT-PCR experiment for each primer pair was determined by constructing a standard curve with serial sample dilutions. Relative gene expression was calculated with comparative Ct method [28] after normalization to beta-glucuronidase (GUSB), an endogenous control, by determining mRNA abundance in each sample relative to the reference sample (calibrator). Outliers were repeated in duplicates and persistent outliers were removed from the final analysis.

### Statistical Analysis

Student's t- test and univariate analysis were used to determine the differences in normalized mRNA expression levels and other quantitative traits between the cases and controls. Normality distribution of mRNA expression levels was assessed using Q-Q plot. Statistical differences for the experiments involving three or more groups were determined using analysis of variance (ANOVA) test. Pearson correlation was performed to evaluate correlations between the biomarkers. Age and gender were considered as potential confounders and appropriately adjusted for during analysis. All the analysis was carried out using SPSS v 17.0 statistical software package. Data was expressed as mean ± standard error of mean (SEM). All the statistical tests were two-sided, with 95% confidence interval (CIs). A nominal p value of 0.05 or less was considered as statistically significant.

## Results

### Network analysis and characterization of the hub nodes

**Figure 2:**
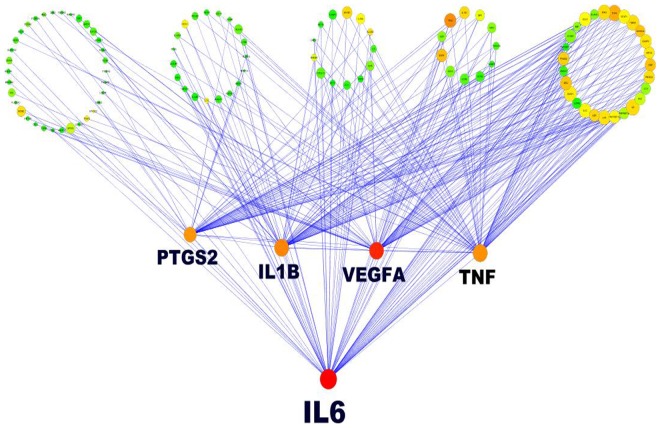
The inflammatory backbone network. The backbone network is derived from a master network consisting of 145 nodes connected via 1234 edges, constructed from 124 combined gene sets obtained from *Polysearch* and *CADgene* database. This backbone network consists of 111 nodes connected via 348 edges. Node color code: shades of red to green color depicts node with highest to lowest value of betweenness centrality (BC); Node size: from biggest to smallest circle map the node degree. Bigger and bright colored nodes represent genes with more links. IL-6 appears to be the super hub gene in the network with largest degree and highest BC.

The extended PPI network generated using the 124 seed genes in *STRING* resulted in 1234 interactions between 145 nodes, of which 21 new nodes were pulled out based on their protein- protein interactions. The network obtained from *STRING* was subsequently analyzed as described in the methodology section. Nodes with large degree and high BC represent the key genes. In the present study, 5% of the nodes had a degree value greater than 50 and 4% of the nodes had BC above 0.05. In total, 3% of the nodes (5 genes) were finally selected having high degree and high BC values as shown in table 1. The network so constructed revealed a scale free architecture with a power-law degree distribution r-squared value of 0.626. Interleukin-6 (IL-6), vascular endothelial growth factor A (VEGFA), interleukin-1 beta (IL1B), tumor necrosis factor (TNF) and prostaglandin-endoperoxide synthase 2 (PTGS2) were the key nodes (hub), displaying the highest connectivity within the network. The backbone network was subsequently constructed using these 5 key genes, which consisted of 111 nodes connected via 348 edges (Figure 2). Here, IL-6 occupied the centre of the backbone network, having the largest degree and highest BC, which suggests that IL-6 could be considered as a super-hub gene.

**Table 1:** Key genes selected based on topological parameters like BC and degree.

Gene	Degree	Betweenness centrality (BC)
IL6	77	0.10995103
VEGFA	61	0.09511043
IL1B	61	0.05885691
TNF	62	0.05394098
PTGS2	49	0.05242533

Node degree and Betweenness Centrality (BC) are topological parameters used for gene prioritization in the network; A cut-off of BC >0.05 and/or node degree >50 were considered for gene prioritization. IL-6 constituted the super hub node having the largest degree and the highest BC.

### Identification of regulators for the hub nodes

**Figure 3:**
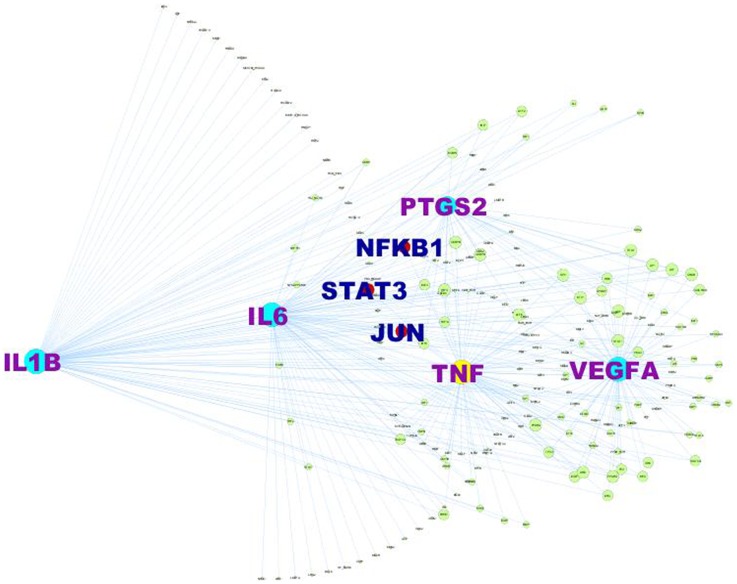
Interactive regulatory network constituted with five hub genes and three common transcription factors. 184 unique transcription factors (TFs) obtained with *MatInspector* were used for the construction of this network with *Cytoscape v 2.8.3*. The TFs showing top connectivity are represented as red circle while the putative target genes are shown in turquoise color. The text has been enlarged for easy identification.

To investigate if there was a common transcriptional regulatory mechanism for these hub genes, we performed transcription promoter analysis and obtained a list of transcription factors using *MatInspector*. Further refinement of TFs based on evidence >1 resulted in 69 TFs belonging to 19 families for IL6, 75 TFs belonging to 18 families for VEGFA, 74 TFs belonging to 21 families for IL1B, 70 TFs belonging to 21 families for TNF and 37 TFs belonging to 10 families for PTGS2. All the TFs were subsequently combined and redundancy was removed to obtain a final list of 184 unique TFs. A regulatory network was constructed by integrating these TFs and their target genes (Figure 3). Nuclear factor kappa B1 (NFKB1), signal transducer and activator of transcription 3 (STAT3) and JUN showed the highest connections suggesting that these regulators could play a critical role in activating these putative inflammatory genes.

### Validation of the hub genes

**Figure 4:**
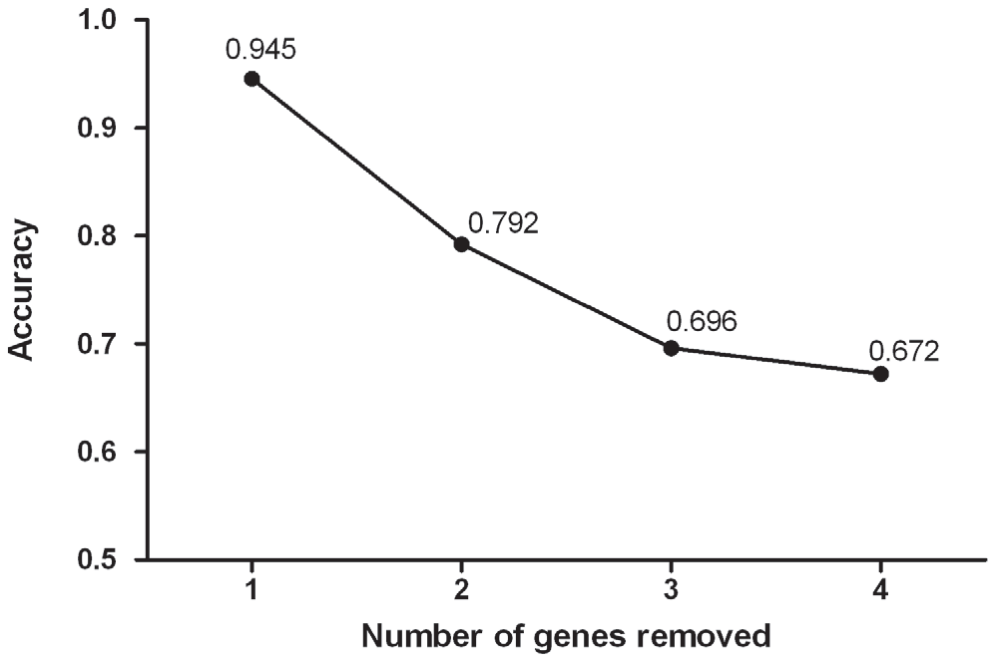
Accuracy of hub genes in the test network. Horizontal axis represents the number of genes removed in the test network. The vertical axis represents accuracy of the respective test network.

We performed cross-validation to confirm if the selected hub genes are being retained even after random gene removal. We ran 97 test networks by randomly removing the top 22 nodes having the highest BC and node degree. By doing so, the overall accuracy of the backbone network was estimated to be 0.7763. The frequency of the hub nodes, IL-6, IL1B, TNF, and PTGS2, was good for 1 to 4 gene deletions. However, the frequency of VEGFA was good for 1-2 gene removals, but decreased with three or more gene removals. The frequency of hub genes and accuracy of the 97 test networks are provided in Table 2 and Figure 4.

**Table 2:** Frequency of the hub genes and the accuracy of the backbone in 97 test network.

Number of removed gene	Frequency of the hub nodes in test network					Accuracy of the backbone	Number of test networks
	IL6	IL1B	TNF	PTGS2	VEGFA		
1	21	21	21	21	20	0.945	22
2	20	20	20	19	20	0.792	25
3	20	23	18	17	9	0.696	25
4	20	23	18	19	4	0.672	25
Overall	81	87	77	76	53	0.776	97

### Molecular function and Pathway analysis

**Figure 5:**
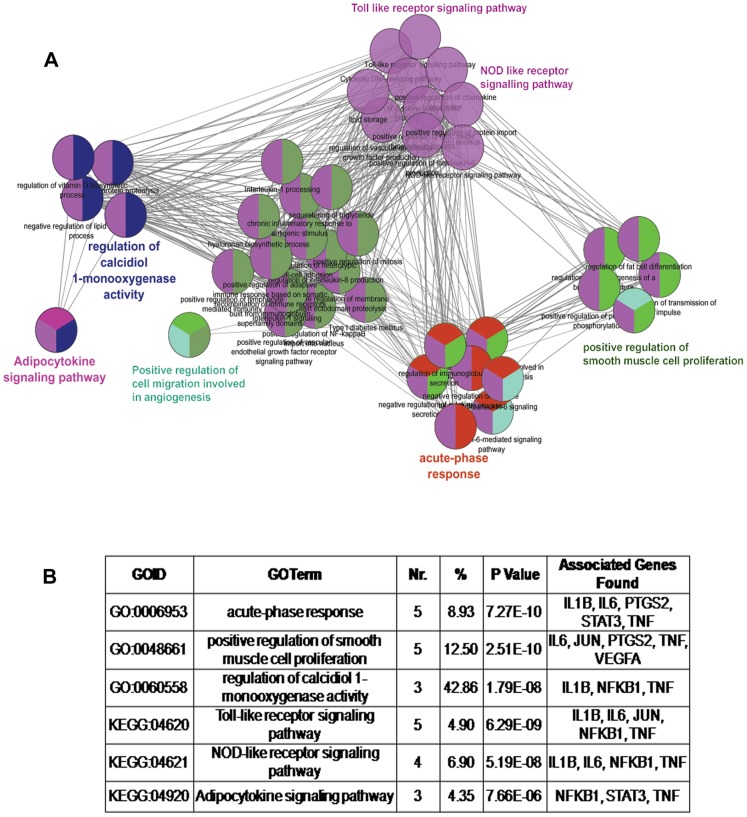
Grouping of network based on functionally enriched GO terms and pathways. **A.** Functionally grouped network of enriched categories was generated for the hub genes and their regulators using *ClueGO*. GO terms are represented as nodes based on their kappa score level (≥0.3). Functionally grouped networks are linked to their biological function, where only the most significant term in the group is labeled. Functionally related groups partially overlap. Visualization has been carried out using *Cytoscape 2.8.3*. **B.** Table provides the results of *ClueGO* analysis. Nr: Number of genes from our list (8-genes) associated with the GO term. %: percentage of genes found from the total number of associated genes. p value: p value of the GO term after Benjamini-Hochberg correction. Associated genes are represented from among those associated with either GO term or specific pathway.

To get an insight on the pathways that the 5 hub genes and their 3 common transcriptional regulators might be involved in, we performed enrichment analysis using *ClueGO* software. Overall, 36 GO terms and 20 pathways were significantly enriched, which could be categorized into six GO groups as represented in Figure 5. The main GO categories were as follows: positive regulation of smooth muscle cell proliferation, acute-phase response and regulation of calcidiol 1-monooxygenase activity. The main pathways from *KEGG* and *REACTOME* belonged to the following categories: toll-like receptor signaling, NOD-like receptor signaling and adipocytokine signaling pathways (Figure 5).

### Clinical characteristics of study subjects

The characteristics of study subjects in the gene expression study are described in Table 3. The average age of cases and controls was around 50 years, with higher frequency of males (89%) than female (11%). Hypertension and diabetes were higher in the cases than in the controls. There were 51 (79.7%) and 45 (70.3%) individuals having MS in cases and controls, respectively. There were 35 cases (62.5%) with chronic stable angina and 21cases (37.5%) with myocardial infarction.

**Table 3:** Clinical characteristics of study participants included in the gene expression study.

Clinical Factors	Cases	Controls	p value
	(N = 64)	(N = 64)	
Age (years)	50.02±0.817	50.22±0.788	0.858
Males N (%)	57(89.1)	57(89.1)	-
Females N (%)	7(10.9)	7(10.9)	-
BMI(kg/m^2^)	25.24±0.416	25.11±0.55	0.847
Waist Circumference (cm)	89.71±1.10	93.54±1.09	0.015
Hip Circumference (cm)	93.95±1.01	94.62±1.25	0.68
Waist/Hip Ratio (cm)	0.95±0.009	0.98±0.010	0.015
SBP (mmHg)	120.75±1.41	124.09±2.49	0.246
DBP (mmHg)	77.84±0.90	79.25±1.97	0.518
Age at onset (years)	49.33±0.934		-
Stable Angina N (%)	35(62.5)		
Myocardial infarction N (%)	21(37.5)		
Number of Diseased Vessel	1 = 7(12.3)		
	2 = 18(31.6)		
	3 = 32(56.1)		
Laboratory studies			
TC (mg/dl)	147.38±5.92	143.95±11.52	2.29*10^−6^
TG (mg/dl)	161.79±8.19	194.71±9.06	0.218
HDL-c (mg/dl)	31.48±1.01	39.89±1.445	7.70*10^−6^
LDL-c (mg/dl)	83.54±5.22	117.99±4.17	8.59*10^−7^
Medical history			
Smoking N (%)	26(40.6)	26(40.6)	-
Hypertension N (%)	30(47.6)	7 (10.9)	5.06*10^−6^
Diabetes mellitus N (%)	27(42.2)	8 (12.5)	0.00028
Metabolic Syndrome N (%)	51 (79.7)	45 (70.3)	0.154
Medications			
Statin N (%)	41 (64%)	-	-

Continuous variables are expressed as mean±standard deviation. BMI, body mass index; SBP, systolic blood pressure; DBP, diastolic blood pressure; TC-Total cholesterol; TG- Triglyceride; HDL-c, High Density Lipoprotein cholesterol; LDL-c, Low Density Lipoprotein cholesterol.

Frequency distribution of subjects based on the number of disease vessels were as follows: 0 or 1 vessel: 7 (12.3%), 2 vessels: 18 (31.6%), 3 or more vessels: 32 (56.1%). TC and LDL-c levels were lower in cases than in the controls, which might be attributed to the higher usage of lipid lowering drugs in the former group.

### Expression analysis of key genes and their regulators

**Figure 6:**
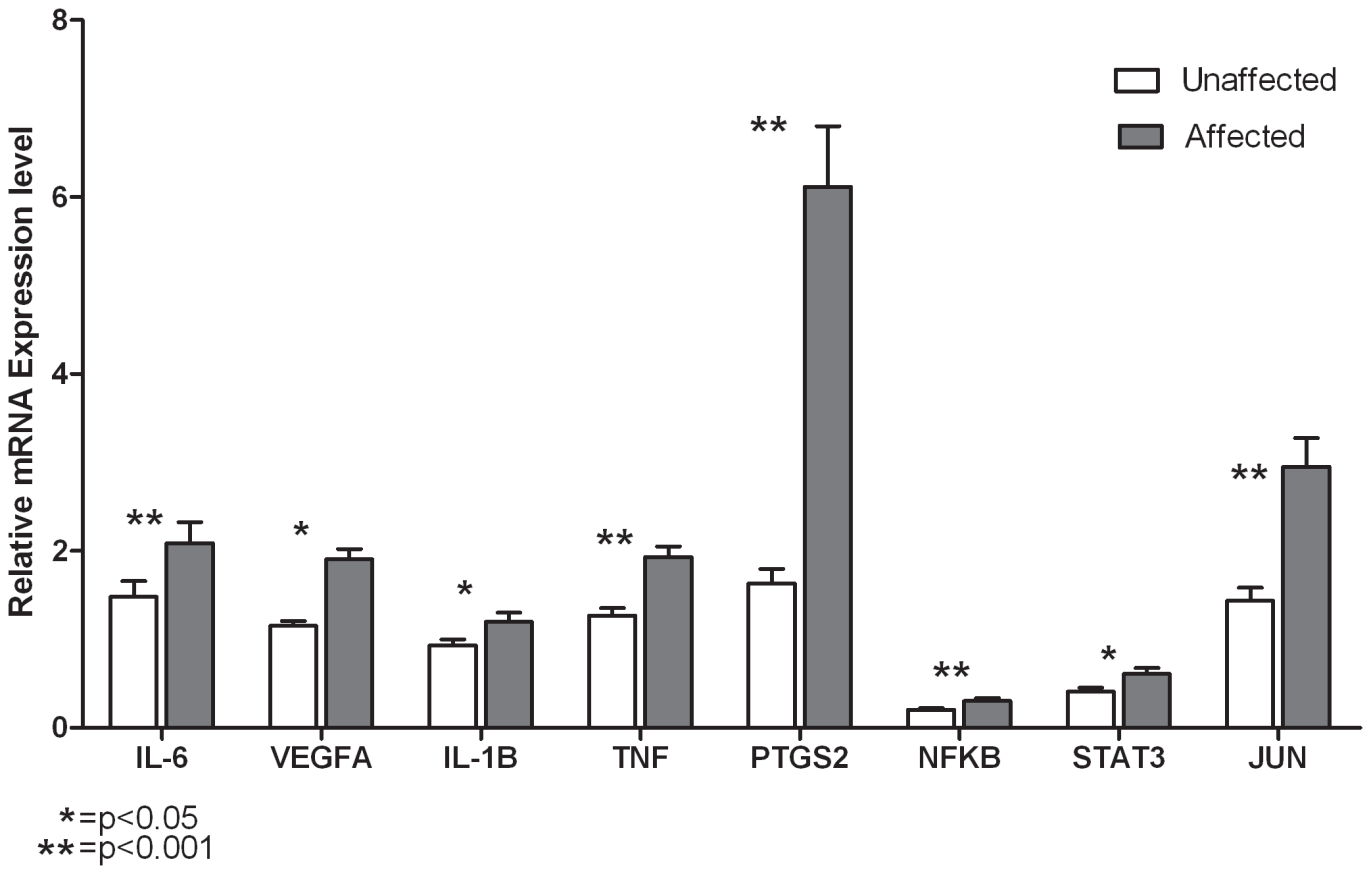
Relative expression of hub genes and their transcription factors. Gene expression of individual samples was normalized to GUSB mRNA levels. The data are expressed as mean ± S.E.M. for the affected (black bar) and unaffected (white bar) subjects.

In order to discern the association of key genes and transcription factors identified through network analysis, we measured the relative expression of all the 5 candidate genes and the 3 regulators in 64 cases and 64 controls. Significant differences were observed in the mean expression levels between cases versus controls for IL6 (2.08±0.23 vs 1.48±0.17; p = 0.02), VEGFA (1.91±0.11 vs 1.15; p<0.001), IL-1B (1.19±0.11 vs 0.93±0.07; p = 0.002), TNF (1.93±0.12 vs 1.26±0.08; p = 0.03), PTGS2 (6.12±0.68 vs 1.63±0.16; p<0.001), NFKB1 (0.31±0.03 vs 0.21±0.02; p<0.001), STAT3 (0.61±0.06 vs 0.41±0.04; p = 0.02) and JUN (2.95±0.32 vs 1.43±0.15; p<0.001) (Figure 6). Statistical significance was retained even after adjusting for age and gender.

Subset analysis of gene expression in individuals with (N = 26) and without (N = 66) diabetes and with (N = 24) and without (N = 66) hypertension showed significant differences for PTGS2 in individuals with diabetes (6.23±0.94 vs 2.93±0.40; p = 0.009) and hypertension (5.83±1.01 vs 3.04±0.39; p = 0.001), respectively. Analysis of these markers in individuals with MS (N = 70) and without MS (N = 24) showed significant expression differences for PTGS2 (4.13±0.51 vs 2.73±0.54; p = 0.008) and VEGFA (1.59±0.09 vs 1.38±0.10; p = 0.006). Furthermore, there was strong correlation in the mean expression levels of these hub genes with each other as well as with the transcriptional regulators (r = 0.24–0.78; p<0.05) (Table S4).

## Discussion

Inflammation plays a pivotal role in atherosclerosis which can be attributed to the release of various enzymes, cytokines and chemokines during the different stages of disease development and progression [29], [30]. In line with this, several inflammatory genes are known to be associated with CAD [4]. Experimental validation of all the potential candidate genes is impractical due to the prohibitive cost and the time involved. In such a scenario, network analysis can serve as a powerful tool for gene prioritization. We have used a network based approach to identify key connecting molecules from a given panel of inflammatory genes. As our understanding of the molecular basis of CAD may suffer from incompleteness of available data, we considered inflammatory genes implicated with different disease types. Studies on human disease network in fact indicate a common genetic origin for many diseases, thus suggesting interconnectedness among the genes [31]. Hence, for this study, 68 inflammatory genes associated with CAD were selected using the *CADgene* database, which was further extended by selecting additional inflammatory genes associated with other diseases. In this manner, the number of genes increased from 68 to 124, which was subsequently used for the construction of the network.

The main network was characterized by a few large nodes which constituted the hub, having many connections and other smaller nodes with fewer connections [32]. This primary network consisted of 1234 interactions between 145 nodes and the relative importance of the genes in the network was determined based on the centrality measures of node degree >50 and BC value >0.05. In this manner, we identified five key genes, IL6, VEGFA, IL-1B, TNF and PTGS2 that were used to construct the backbone network with an aim to analyze the interconnectivity of the hub genes with the other genes. The backbone interaction network included 111 nodes connected via 348 edges and implied that 76% of the connections were formed due to the five hub nodes, which may play a critical role in the inflammatory-driven disease process. In fact, IL-6 formed the central molecule, showing the highest connectivity and constituting a super-hub in relation to the network. According to the centrality-lethality rule, hubs are more likely to be functionally relevant than the other genes [33]. In fact, it has been demonstrated that deletion of the hub genes produce a higher frequency of sick phenotypes than the deletion of genes showing low connectivity [34]. These topological properties of the network have been employed to improve our understanding of key genes associated with the diseases. For instance, Sarajlic et al employed network topological features to predict novel cardiovascular genes that are enriched in drug targets and driver genes [11]. As explained in the introduction section, inflammation plays a pivotal role in CAD and network biology approaches also postulate that inflammation and immune response related genes have yielded the most consistent signals across different diseases [35]. Zhang et al. applied network topological features to construct a unified classifier and then used it to predict the candidate genes for CAD and their results showed an overrepresentation of the genes belonging to the inflammatory pathway [12]. Through an effective analysis of the genome wide action of liver X receptor α (LXRα) in foam cells and macrophages using chromatin immunoprecipitation sequencing, gene expression and *in vitro* experimentation, complimented by transcriptional network analysis, Feldmann et al, identified highly integrated LXRα ligand-dependent transcriptional networks that contributed to the reversal of cholesterol efflux and reduced inflammation, thus resulting in effective prevention of atherogenesis [15]. In yet another recent report on the molecular processes occurring on the atherosclerotic arterial wall in response to plasma cholesterol lowering therapies, distinct differences were observed in the type of transcription factors and associated regulatory networks of plasma cholesterol lowering-responsive gene sets in early, mature, and advanced lesions [16]. These studies highlight the effectiveness of combing experimentation with network analysis to delineate the complexity of the atherosclerotic disease process.

We performed quantitative validation of the causal genes followed by functional enrichment analysis. Literature validations of hub genes have also confirmed their role in the pathogenesis of CAD and the details are discussed in the ensuing paragraphs. Next, in order, to evaluate the robustness of our network and eliminate false positives and false negatives, we constructed 97 test networks wherein we found that the frequency of the hub genes was not grossly altered. Concurrent with our findings, other studies have also demonstrated that the scale free network exhibit certain level of tolerance against errors, implying that the PPI network topologies are robust with respect to perturbations [36].

Inflammatory response results from a complex interplay between the different inflammatory molecules which act either in an autocrine or paracrine manner to induce acute phase response. The inflammatory cascade is initiated by the pro-inflammatory cytokines, IL1B and TNF, which induce IL-6 synthesis, mediated by Phosphatidylinositol 3-Kinase-dependent AKT/IκB Kinase α Pathway targeting AP-1 (Activator Protein-1) [37]. Cholesterol crystals in the plaque activate IL-1B secretion in the macrophages and thus link cholesterol metabolism to inflammation [38]. Increased circulatory levels of IL-1β in patients with unstable angina indicates its contribution to the acute disease process [39]. Clinical studies have also shown increased expression of IL-1B and TNF in the atherosclerotic plaques [40], [41]. Consistent with these reports, we also observed increased levels of IL-1B and TNF in the affected cases than in the asymptomatic controls. Studies in mouse models however, suggest a dual role for IL1B, wherein absence of IL-1B decreases the severity of atherosclerosis [42]. On the other hand, Alexander et al have shown that the inactivation of IL-1B enhance atherosclerotic plaque stability [43]. In the present study, IL-6 was represented at the centre of the backbone network, which highlights their significance as a pivotal player in the CAD inflammatory network. In fact, in a study on the IARS cohort, we have previously shown significant association between IL-6 gene polymorphisms and premature CAD and that IL6 acts as a key regulator of acute phase reactants, namely hsCRP and fibrinogen in the IARS cohort [44].

Vascular endothelial growth factor A is a key member of the family of growth factors. It is involved in the angiogenesis and is essential for tissue growth as well as organ repair process [45]. Higher circulating levels of VEGFA have been previously detected in serum of patients with cardiovascular disease [46], [47]. The enzyme PTGS2 [more commonly known as cyclooxygenase-2 (COX-2)] mediates the production of prostaglandins, which can induce an inflammatory response [48]. PTGS2 has been shown to promote plaque rupture through the metalloproteases in symptomatic plaques showing recent evidence of ischemic attack [49]. Other studies have also reported a strong correlation between COX-2 and VEGFA, which together play an important role in angiogenesis [50]. We noted increased expression and positive correlation of PTGS2 and VEGFA in the present study.

Controlled regulation of the gene transcripts is essential for maintaining homeostasis and a disease free state. Since specific TFs bind to the promoter region of their gene targets to regulate expression, we analyzed the regulatory network to understand the pattern of relationship between the five hub genes and their specific TFs. From among the 184 unique TFs, we identified NFKB1, JUN and STAT3 as the three most common factors, whose role in CAD is well documented [51]–[53]. Recently, the CARDIoGRAMplusC4D consortium carried out a large scale association study followed by network analysis and identified NF-κB, MAPK and JAK-STAT as the key signaling pathways involved in the pathogenesis of CAD [8]. Inhibition of NFKB has been shown to retard atherosclerotic disease progression in apoE/LDLR double knockout mice models [54]. In fact, the NFKB family as a whole plays a crucial role in regulating a number of processes in the cardiovascular system such as inflammation, cell survival, cell proliferation, cellular response to stress, hypoxia and ischemia [55]. Among the many transcriptional targets for NFKB, IL6, VEGFA, IL-1B, TNF and PTGS2 are considered to be important and regulate the cytokines, chemokines and the adhesion molecules [53]. JUN is an important protein constituent of AP-1 (activator protein-1) transcription factors wherein, the c-Jun protein forms a homo or heterodimer with c-Fos, resulting in the AP-1 transcription factor complex [56]. The role of c-Jun in cellular proliferation, differentiation and apoptosis is well established [57], [58]. Studies also suggest that c-Jun can regulate neo intima formation and promote the proliferation and differentiation of vascular smooth muscle cells [59] and human endometrial cells [60]. AP-1 has been shown to be involved in the activation of matrix metalloproteinase (MMP) in progressive and unstable atherosclerotic plaques [61] as well as during the early disease process [52]. JUN appeared to be one of the most significant transcription factors in our regulatory network.

STAT3 has been implicated in the cardiovascular inflammatory process [51]. STAT3 is activated by phosphorylation of tyrosine 705 and serine 727 in response to the various cytokines and growth factors, including IL-6, IL-10, epidermal growth factor (EGF), interferon-alpha (IFN-alpha) and interferon-gamma (IFN-gamma) in distinct patterns [62], [63]. In addition, STAT3 is activated under a variety of stress conditions [64], translocate to the nucleus, where they bind to specific DNA sequences and regulate the expression of target genes. Initially, STAT3 was thought to be an acute phase reactant [65]. However, recent studies have shown that it exerts a cardio protective effect [66]. Experimental studies in rats have shown elevated IL6 expression in acute MI, accompanied by a marked increase in STAT3 through the activation of the JAK/STAT pathway [67], [68]. Our results are in line with the earlier reports, wherein both IL6 and STAT3 showed higher expression in CAD patients relative to the controls. We observed strong positive correlation between STAT3 and VEGFA (r = 0.565; p<0.001) which is comparable to the study by Osugi et al where they have shown that activation of STAT3 in the cardiac myocytes increased VEGF expression [69]. In fact, STAT3 activation could be ascribed to a protective systemic response to reduce cardiac damage and promote active remodeling. The above studies demonstrate the critical role of STAT3 as a negative regulator of inflammation and its potential as a therapeutic target for atherosclerosis.

Through functional enrichment, we identified the following pathways associated with the hub genes and their regulators: smooth muscle cell proliferation, acute-phase response, calcidiol 1-monooxygenase activity, toll-like receptor (TLR), NOD-like receptor (NLR) and adipocytokine signaling pathways. Numerous studies have demonstrated the involvement of these biological processes in atherosclerosis. Abnormal vascular smooth muscle cell proliferation has been shown to increase the production of acute-phase reactant proteins like CRP, while Vitamin D deficiency has been implicated in cardiovascular disease [70]–[72]. Both TLR and NLR signaling pathways belong to a specific family of pattern recognition receptors that trigger the expression of several genes involved in innate immune response [73]. TLR signaling is associated with chronic inflammatory response [74] while NLR signaling results in the formation of inflammasomes, which along with TLR, orchestrate the induction of IL-1B and IL-18 secretion [75]. TLR-1, TLR-2 and TLR-4 have been shown to be elevated in human atherosclerotic lesions [76]. In a pilot study, we have previously shown the increased expression of TLR2 in the peripheral whole blood of CAD subjects [77]. Among NLRs, NLR-related protein 3 is one of the better characterized receptors in atherosclerosis. Interestingly, there have been studies to show that cholesterol crystals which are characteristic of atherosclerotic lesions, activate NLR3 and promote the secretion of pro-inflammatory cytokines [38], [78]. Adipocytokines are biologically active molecules secreted by the adipose tissue and promote the development of obesity-mediated atherosclerotic disease. Adipose tissue from obese individuals synthesizes and releases leptin, adiponectin and pro-inflammatory molecules such as TNF, IL1B and IL-6 that increase the cardiovascular risk [79].

We recognize certain limitations in our study. The network generated was based on information obtained from literature mining and hence could carry an inherent bias. The generated network might also suffer from incompleteness or missing interactions since they have been derived from published reports and our understanding of cardiovascular genetics is far from complete. However, we have addressed these issues to some extent by using two different data sources to arrive at a comprehensive panel of inflammatory genes.

In conclusion, we have identified IL-6, VEGFA, IL1B, TNF and PTGS2 genes to play a pivotal role in the inflammation-driven atherosclerotic disease process from among a vast array of putative inflammatory genes using network-based analysis. Of the associated transcriptional regulatory factors, NFKB1, STAT3 and JUN was shown to highly network with the above gene targets and provide a glimpse of the inherent complexity of the inflammatory bionetwork. The frequency of the hub genes was also found to be unaffected against the changes in the initial seed genes. We were finally able to confirm our hypothesis with a pilot case-control gene expression association study. While there is convincing evidence to show an active role for these candidate genes and their transcription factors based on *in vitro* and *in vivo* experimental studies in animal models and humans, we have shown comparable outcomes using a network based approach. The advantage lies in our ability to visualize not only complex interactions among the individual components but also comprehend their relative importance in the network based on well defined topological parameters. Such visualization and identification can promote a better understanding of the underlying disease process and also identify specific gene targets for therapy. Nonetheless, additional studies are required to confirm these initial findings and to eventually realize their true potential in a clinical setting.

## Supporting Information

Table S1 **List of genes extracted from** ***Polysearch*** **and** ***CADgene*** **database that are associated with inflammation.** Relevancy score has been provided for all the genes selected from *Polysearch*. Other genes have been selected from *CADgene* database.
(DOCX)

Table S2 **Oligonucleotides used for SYBR Green RT-qPCR.** Primers were selected from PrimerBank and the respective IDs have been provided.
(DOCX)

Table S3 **Detailed information about frequency of hub node and accuracy of the backbone in the 97 test networks.**
(XLSX)

Table S4 **Representation of correlation matrix for expression of candidate inflammatory genes.** Pearson's correlation between the transcriptome is displayed in a color gradient of red to green, where red indicates high correlation and green indicates least correlation. Range of red to orange color indicates positive correlation. * indicates p<0.05; correlation coefficient range was 0.249 to 0.79.
(DOCX)
