# Supplementary material for: Network Analysis of Inflammatory Genes and Their Transcriptional Regulators in Coronary Artery Disease
Source: PLoS One. 2014 Apr 15;9(4):e94328. doi: 10.1371/journal.pone.0094328 (PMC3988072; doi:10.1371/journal.pone.0094328)
Supplement: Table S1 — List of genes extracted from Polysearch and CADgene database that are associated with inflammation. Relevancy score has been provided for all the genes selected from Polysearch. Other genes have been selected from CADgene database. (DOCX) [file pone.0094328.s001.docx]

**Table S1. List of genes extracted from *Polysearch* and *CADgene* database that are associated with inflammation**

| **Sl No.** | **Symbol** | **Description** | **Z score** |  | **Sl No.** | **Symbol** | **Description** | **Z score** |
| --- | --- | --- | --- | --- | --- | --- | --- | --- |
| 1 | ABCB1 | ATP-binding cassette, sub-family B (MDR/TAP), member 1 | 2.5 |  | 63 | IL1RN | interleukin 1 receptor antagonist |  |
| 2 | ABRA | Actin binding Rho activating protein | 1.1 |  | 64 | IL5 | interleukin 5 | 1.3 |
| 3 | ADAM17 | ADAM metallopeptidase domain 17 |  |  | 65 | IL8 | interleukin 8 | 7.3 |
| 4 | ADIPOQ | adiponectin | 3.9 |  | 66 | IRF5 | interferon regulatory factor 5 |  |
| 5 | ADIPOR2 | adiponectin receptor 2 |  |  | 67 | KLF2 | Kruppel-like factor 2 |  |
| 6 | ALOX15 | arachidonate 15-lipoxygenase |  |  | 68 | LEP | leptin | 3.6 |
| 7 | ALOX5 | arachidonate 5-lipoxygenase |  |  | 69 | LGALS2 | lectin, galactoside-binding soluble 2 |  |
| 8 | ALOX5AP | arachidonate 5-lipoxygenase-activating protein |  |  | 70 | LGALS3 | lectin, galactoside-binding soluble 3 | 2.7 |
| 9 | AMPK | 5' AMP activated protein kinase catalytic subunit beta 1 | 1.4 |  | 71 | LTA | lymphotoxin alpha |  |
| 10 | ANGPT1 | Angiopoietin 1 | 1.8 |  | 72 | LTA4H | leukotriene A4 hydrolase |  |
| 11 | ANGPT2 | Angiopoietin 2 | 1.1 |  | 73 | MAPK | mitogen-activated protein kinase 1 | 3.5 |
| 12 | AGT | Angiotensin | 1.5 |  | 74 | MBD2 | methyl-CpG binding domain protein 2 | 1.6 |
| 13 | ARL15 | ADP-ribosylation factor-like protein 15 |  |  | 75 | MBL2 | mannose-binding lectin (protein C) 2 |  |
| 14 | BDKRB2 | bradykinin receptor B2 |  |  | 76 | MIF | macrophage migration inhibitory factor |  |
| 15 | C Rel | C-Rel proto-oncogene protein | 1 |  | 77 | MPO | myeloperoxidase | 1.1 |
| 16 | c Src | proto-oncogene **c**-**Src** | 1 |  | 78 | NAMPT | nicotinamide phosphoribosyltransferase | 1 |
| 17 | CASP1 | caspase 1 |  |  | 79 | NAP 1 | napsin A aspartic peptidase | 1.6 |
| 18 | CASP3 | caspase 3 | 1.6 |  | 80 | NFKB1 | nuclear factor of kappa light polypeptide gene enhancer in B-cells 1 |  |
| 19 | CB1 | cannabinoid receptor 1 | 1.1 |  | 81 | NFKBIL1 | nuclear factor of kappa light polypeptide gene enhancer in B-cells inhibitor-like 1 |  |
| 20 | CCL11 | chemokine (C-C motif) ligand 11 |  |  | 82 | NOD1 | nucleotide-binding oligomerization domain containing 1 |  |
| 21 | CCL2 | chemokine (C-C motif) ligand 2 |  |  | 83 | NOD2 | nucleotide-binding oligomerization domain containing 2 |  |
| 22 | CCL5 | chemokine (C-C motif) ligand 5 |  |  | 84 | NR1H3 | nuclear receptor subfamily 1, group H, member 3 |  |
| 23 | CCR2 | chemokine (C-C motif) receptor 2 |  |  | 85 | NR4A2 | nuclear receptor subfamily 4, group A, member 2 |  |
| 24 | CCR5 | chemokine (C-C motif) receptor 5 (gene/pseudogene) |  |  | 86 | PDE4D | phosphodiesterase 4D |  |
| 25 | CD14 | CD14 molecule |  |  | 87 | PI3K | Phosphatidylinositol 3 kinase regulatory subunit alpha | 1.3 |
| 26 | CD45 | protein tyrosine phosphatase, receptor type, C | 1.6 |  | 88 | PLA2G10 | phospholipase A2 group X |  |
| 27 | CD74 | CD74 molecule, major histocompatibility complex, class II invariant chain | 3.2 |  | 89 | PON1 | paraoxonase 1 | 2.1 |
| 28 | CD93 | CD93 molecule |  |  | 90 | PPARG | peroxisome proliferator-activated receptor gamma | 1.7 |
| 29 | CFH | complement factor H |  |  | 91 | PSMA6 | proteasome subunit alpha type 6 |  |
| 30 | CHI3L1 | chitinase 3-like 1 (cartilage glycoprotein-39) |  |  | 92 | PTGER2 | prostaglandin E receptor 2 |  |
| 31 | CRP | C reactive protein | 12.2 |  | 93 | PTGS2 | prostaglandin-endoperoxide synthase 2 |  |
| 32 | CSF-1 | macrophage colony stimulating factor | 1.8 |  | 94 | PTX3 | pentraxin 3 | 3.2 |
| 33 | CX3CR1 | chemokine (C-X3-C motif) receptor 1 |  |  | 95 | KLK3 | Prostate-specific antigen | 1.1 |
| 34 | CXCL12 | chemokine (C-X-C motif) ligand 12 |  |  | 96 | RAGE | Advanced glycosylation end product-specific receptor | 2.4 |
| 35 | CXCL16 | chemokine (C-X-C motif) ligand 16 |  |  | 97 | RETN | resistin | 1.2 |
| 36 | DED | apoptosis antagonizing transcription factor | 1.2 |  | 98 | S100B | S100 calcium binding protein B | 5.4 |
| 37 | ELANE | Neutrophil elastase |  |  | 99 | S100A12 | S100 calcium binding protein A12 | 2.3 |
| 38 | ESR1 | estrogen receptor alpha | 1.8 |  | 100 | S100A8 | S100 calcium binding protein A8 | 2.2 |
| 39 | FAM89A | family with sequence similarity 89, member A | 1.4 |  | 101 | SELS | Selenoprotein S |  |
| 40 | FCGR2A | Fc fragment of IgG, low affinity IIa, receptor (CD32) |  |  | 102 | SFTPD | surfactant protein D |  |
| 41 | FN1 | fibronectin 1 |  |  | 103 | SIGIRR | single immunoglobulin and toll-interleukin 1 receptor (TIR) domain | 3.2 |
| 42 | GPCR | G-protein coupled receptor | 1.7 |  | 104 | SIRT1 | sirtuin 1 | 5.4 |
| 43 | HIF1A | Hypoxia inducible factor 1alpha | 3.3 |  | 105 | SOD | superoxide dismutase | 2.2 |
| 44 | HLA-B | major histocompatibility complex, class I, B | 3.2 |  | 106 | SP1 | Sp1 transcription factor | 1.2 |
| 45 | HMGB1 | high mobility group box 1 | 2.7 |  | 107 | SPP1 | secreted phosphoprotein 1 | 1.9 |
| 46 | HMOX1 | Heme Oxygenase 1 | 2.5 |  | 108 | ST2 | suppression of tumorigenicity 2 | 1.3 |
| 47 | HSPA2 | heat shock 70kDa protein 2 |  |  | 109 | TERT | telomerase reverse transcriptase | 2.4 |
| 48 | CD47 | CD47 molecule | 1..3 |  | 110 | TGFB1 | transforming growth factor beta 1 |  |
| 49 | ICAM1 | intercellular adhesion molecule 1 |  |  | 111 | TLR4 | toll like receptor 4 | 3.7 |
| 50 | IFNG | interferon gamma |  |  | 112 | TNF | tumor necrosis factor |  |
| 51 | IL10 | interleukin 10 | 5.9 |  | 113 | TNFAIP2 | tumor necrosis factor, alpha-induced protein 2 | 1.4 |
| 52 | IL17 | interleukin 17 | 2.6 |  | 114 | TNFAIP3 | tumor necrosis factor, alpha-induced protein 3 |  |
| 53 | IL21 | interleukin 21 | 2.3 |  | 115 | TNFAIP8 | tumor necrosis factor, alpha-induced protein 8 | 1.2 |
| 54 | IL23 | interleukin 23 | 2.4 |  | 116 | TNFRSF11B | tumor necrosis factor receptor superfamily, member 11b |  |
| 55 | IL4 | interleukin 4 | 3.4 |  | 117 | TNFRSF1A | tumor necrosis factor receptor superfamily, member 1A |  |
| 56 | IL6 | interleukin 6 | 15.2 |  | 118 | TNFRSF1B | tumor necrosis factor receptor superfamily, member 1B |  |
| 57 | IL12B | interleukin 12B |  |  | 119 | TNFRSF4 | tumor necrosis factor receptor superfamily, member 4 |  |
| 58 | IL18 | interleukin 18 | 2.9 |  | 120 | TNFSF11 | tumor necrosis factor (ligand) superfamily, member 11 |  |
| 59 | IL1A | interleukin 1 alpha |  |  | 121 | TRPV4 | transient receptor potential cation channel, subfamily V, member 4 | 1.3 |
| 60 | IL1B | interleukin 1 beta |  |  | 122 | TSLP | Thymic Stromal Lymphopoietin | 1.6 |
| 61 | IL1R1 | interleukin 1 receptor, type I |  |  | 123 | VCAM1 | vascular cell adhesion molecule 1 | 2.5 |
| 62 | IL1RAP | IL 1 receptor accessory protein | 2.4 |  | 124 | VEGFA | vascular endothelial growth factor | 6.9 |

Relevancy score has been provided for all the genes selected from *Polysearch*. The other genes have been selected from *CADgene* database.
