# Supplementary material for: Network Analysis of Inflammatory Genes and Their Transcriptional Regulators in Coronary Artery Disease
Source: PLoS One. 2014 Apr 15;9(4):e94328. doi: 10.1371/journal.pone.0094328 (PMC3988072; doi:10.1371/journal.pone.0094328)
Supplement: Table S2 — Oligonucleotides used for SYBR Green RT-qPCR. Primers were selected from PrimerBank and the respective IDs have been provided. (DOCX) [file pone.0094328.s002.docx]

**Table S2. Oligonucleotides used for SYBR Green RT-qPCR**

| **Gene** | **Forward primer ( 5'-3')** | **Reverse (5'-3')** | **PrimerBank ID** |
| --- | --- | --- | --- |
| IL-6 | ACTCACCTCTTCAGAACGAATTG | CCATCTTTGGAAGGTTCAGGTTG | 224831235c1 |
| IL-1B | ATGATGGCTTATTACAGTGGCAA | GTCGGAGATTCGTAGCTGGA | 27894305c1 |
| VEGFA | AGGGCAGAATCATCACGAAGT | AGGGTCTCGATTGGATGGCA | 284172466c1 |
| PTGS2 | TAAGTGCGATTGTACCCGGAC | TTTGTAGCCATAGTCAGCATTGT | 223941909c2 |
| NFκB | GAAGCACGAATGACAGAGGC | GCTTGGCGGATTAGCTCTTTT | 259155300c2 |
| STAT3 | ACCAGCAGTATAGCCGCTTC | GCCACAATCCGGGCAATCT | 47080104c2 |
| JUN | TCCAAGTGCCGAAAAAGGAAG | CGAGTTCTGAGCTTTCAAGGT | 44890066c1 |

The primers were selected from PrimerBank and the respective IDs have been provided.
