# Supplementary material for: Network Analysis of Inflammatory Genes and Their Transcriptional Regulators in Coronary Artery Disease
Source: PLoS One. 2014 Apr 15;9(4):e94328. doi: 10.1371/journal.pone.0094328 (PMC3988072; doi:10.1371/journal.pone.0094328)
Supplement: Table S4 — Representation of correlation matrix for expression of candidate inflammatory genes. Pearson's correlation between the transcriptome is displayed in a color gradient of red to green, where red indicates high correlation and green indicates least correlation. Range of red to orange color indicates positive correlation. * indicates p<0.05; correlation coefficient range was 0.249 to 0.79. (DOCX) [file pone.0094328.s004.docx]

**Table S4. Representation of correlation matrix for expression of candidate inflammatory genes**

|  | IL1B | IL6 | TNF | VEGFA | PTGS2 | NFKB1 | STAT3 | JUN |
| --- | --- | --- | --- | --- | --- | --- | --- | --- |
| IL1B | 1 | 0.40* | 0.35* | -.077 | 0.38* | 0.25* | -.14 | -.11 |
| IL6 | 0.40* | 1 | 0.45* | 0.16 | 0.17 | .04 | -.031 | .128 |
| TNF | 0.35* | 0.45* | 1 | 0.27* | .394* | .04 | .101 | .162 |
| VEGFA | -0.08 | 016 | 0.27* | 1 | 0.36* | 0.04* | 0.57* | 0.79* |
| PTGS2 | 0.38* | .17 | 0.39* | 0.36* | 1 | .08 | .16 | 0.30 |
| NFKB1 | 0.25* | 0.04 | 0.043 | 0.04 | 0.08 | 1 | 0.14 | -0.01 |
| STAT3 | -0.14 | -0.03 | .10 | 0.57* | 0.16 | 0.14 | 1 | 0.37* |
| JUN | -0.11 | 0.13 | 0.16 | 0.79* | 0.30* | -0.01 | 0.37* | 1 |

Pearson’s correlation between the transcriptome is displayed in a color gradient of red to green, where red indicates high correlation and green indicate least correlation. Range of red to orange color indicates positive correlation. * indicates p<0.05; correlation coefficient range was 0.249 -0.79.
